# A mosaic genetic structure of the human population living in the South Baltic region during the Iron Age

**DOI:** 10.1038/s41598-018-20705-6

**Published:** 2018-02-06

**Authors:** Ireneusz Stolarek, Anna Juras, Luiza Handschuh, Malgorzata Marcinkowska-Swojak, Anna Philips, Michal Zenczak, Artur Dębski, Hanna Kóčka-Krenz, Janusz Piontek, Piotr Kozlowski, Marek Figlerowicz

**Affiliations:** 1 Institute of Bioorganic Chemistry, Polish Academy of Sciences, Poznan, Poland; 2 Institute of Anthropology, Faculty of Biology, Adam Mickiewicz University, Poznan, Poland; 3 Institute of Archaeology, Collegium Historicum, Adam Mickiewicz University, Poznan, Poland; 4 Institute of Computing Sciences, Poznan University of Technology, Poznan, Poland

## Abstract

Despite the increase in our knowledge about the factors that shaped the genetic structure of the human population in Europe, the demographic processes that occurred during and after the Early Bronze Age (EBA) in Central-East Europe remain unclear. To fill the gap, we isolated and sequenced DNAs of 60 individuals from Kowalewko, a bi-ritual cemetery of the Iron Age (IA) Wielbark culture, located between the Oder and Vistula rivers (Kow-OVIA population). The collected data revealed high genetic diversity of Kow-OVIA, suggesting that it was not a small isolated population. Analyses of mtDNA haplogroup frequencies and genetic distances performed for Kow-OVIA and other ancient European populations showed that Kow-OVIA was most closely linked to the Jutland Iron Age (JIA) population. However, the relationship of both populations to the preceding Late Neolithic (LN) and EBA populations were different. We found that this phenomenon is most likely the consequence of the distinct genetic history observed for Kow-OVIA women and men. Females were related to the Early-Middle Neolithic farmers, whereas males were related to JIA and LN Bell Beakers. In general, our findings disclose the mechanisms that could underlie the formation of the local genetic substructures in the South Baltic region during the IA.

## Introduction

One of the consequences of the rapid development of DNA isolation, enrichment and sequencing technologies is currently observed: the substantial increase of our knowledge about the prehistory of anatomically modern humans (AMH). The first projects that focused on ancient DNA (aDNA) isolated from singular individuals gave a rough idea of when the first representatives of AMH appeared in Africa and how global human expansion occurred. Currently, the genetic studies on a population scale are a pressing need. Together with archaeological and anthropological research, genetic studies can provide new insight into the natural history of *Homo sapiens sapiens*. So far, such population scale studies have most frequently been concentrated on mitochondrial DNA (mtDNA) due to its high copy number, small size and lack of recombination. Consequently, the analyses of ancient mtDNA substantially contribute to unraveling the history of human settlement around the world.

The genetic structure of the European population has been shaped by a series of consecutive or partly concurrent processes that form the following chain of events: (i) Europe colonization by AMH during the Upper Paleolithic Period; (ii) the Late Glacial and Post-glacial recolonization; (iii) introduction of agriculture (Neolithization); (iv) genetic continuation through the Middle Neolithic (MN) and the progressive intermix of the Early European Farmers (EEF) with endogenous hunters-gatherers (HG); (v) the inflow of new genetic components from the southwest and southeast to Central Europe in the Late Neolithic (LN), and formation of separate cultures in the Early Bronze Age (EBA)^1–4^.

The initial colonization of Europe by AMH began approximately forty-five thousand years ago (tya)^5–7^. This colonization was connected to the extinction of *Homo sapiens neanderthalensis* and a spread of AMH HG. One of the characteristics of the HG populations was the domination of mtDNA haplogroup U represented mainly by U2, U4, U5a, U5b, and U8^1,8^. However, the Last Glacial Maximum (LGM), dated 27–16 tya, forced populations living in north Europe to leave this region. Approximately 19 tya, together with climate warming, a recolonization of Europe by indigenous HG was initiated^5,7^. They came from southwestern and eastern refugia where they survived the ice age. Interestingly, approximately 14.5 tya indigenous HG were replaced by the new incoming people, who diverged from the ancestral HG population prior to the beginning of the LGM (29 tya)^9^. Another major inflow of AMH to Europe occurred during the so-called Neolithic revolution, approximately 12 tya^1,4^. This period is inextricably linked with the introduction of agriculture (cultivation and domestication of animals) and a change from a nomadic to sedentary lifestyle. The Neolithic revolution, the so-called “Neolithization”, most likely proceeded in two ways^10^. First, it resulted from cultural changes initiated in the Mediterranean region that spread towards the southwest part of the continent^3,11^. Second, it was connected with the inflow of the EEF from Central Anatolia along the Danube and towards Central Europe^6,12,13^.

Neolithization was accompanied by the introduction of new mtDNA haplogroups (N1a, T2, K, J, HV, V, W, and X), currently called the “Neolithic package”^14^. Interestingly, during the MN, an increase in the occurrence of haplogroups typical for the HG was observed throughout Europe^2,15,16^. In the central part of the continent this phenomenon is believed to be connected with the expansion of the population associated with the Funnel Beaker Culture (TRB ger. Trichterrandbecherkultur)^1,17^. The TRB was mainly spread within the northern areas of Central Europe and Scandinavia.

Next, two events that significantly affected the mtDNA structure in Europe occurred in the LN. They both coexisted for approximately 300 years across Central Europe. The first one was a migration of the Yamnaya steppe herders from the Eurasian Steppe^15^. A consequence of the inflow was the formation and spread of the Corded Ware Culture (CWC)^15,18^ and the occurrence of new mtDNA haplogroups (I, U2, T1, R) in Central and Eastern Europe^2^. The second event was the formation of the Bell Beaker Culture (BBC), most likely in Western Europe, and its propagation in Central Europe^2^. The most significant differences that have been identified so far between the BBC and CWC populations at the mtDNA level are in the lower frequency of I and U2 haplogroups and the significant prevalence of haplogroup H in BBC^2^. Other substantial changes in the mtDNA haplogroup frequency were found in the population associated with the Unetice Culture (UC) that appeared in Central Europe during the EBA; thus, right after the LN. The UC replaced the BBC and CWC; however, its population did not seem to be a direct genetic continuity of the population associated with the two former cultures. The analysis of mtDNA indicated closer similarity of the UC to CWC rather than to BBC^2^.

Considering the above history, one can expect that the genetic profile of mtDNA of the present-day Central Europe populations should be especially highly influenced by the population related to the UC. However, this is not the case. The analysis of contemporary mtDNA showed that the population associated with the BBC had a more considerable impact on the genetic landscape of present-day Central Europe than the populations associated with the eastern CWC and UC^2^. This was despite the latter being chronologically closer to contemporary times than the BBC. These findings suggest that after the EBA, there were significant demographic changes in Central Europe and, consequently, changes in the genetic structure of the Central Europe population. To elucidate this problem, more detailed studies of the populations living in Central Europe between the EBA and present day are necessary. These studies require careful selection of samples with reference not only to time but also to geographical distribution. First, when the contemporary genetic structure of Central and Eastern Europe was formed will be identified. Presently, it is unknown whether this genetic structure was finally shaped by prehistorical or Early Medieval events. The second issue concerns the representativeness of the samples used to define the genetic structure of the Central European population in the EBA and after this period. Thus far, most of the ancient mtDNA analyses were based on samples from Mittelelbe-Saale in Saxony-Anhalt. Genetic studies of the prehistory of populations inhabiting the region of contemporary Poland are sparse. They are based on a very limited number of samples and focused usually on fragments of mtDNA^19–21^.

To obtain a more thorough view of the demographic processes that affected the genetic profile of Central European populations after EBA, we analyzed mtDNA extracted from individuals living in the area between Oder and Vistula rivers (east of Saxony) at the time of the Iron Age (IA). From several burial grounds, we selected a particularly rich and well-characterized one, located in central Wielkopolska (also referred to as Greater Poland) in Kowalewko, near Poznan (52°35′24″N 16°46′44″E). According to the archaeological and anthropological studies, approximately 500 people who had lived on that area from 1 until 200 A.D. were buried there. Most of the burial sites have previously been thoroughly examined archaeologically and anthropologically^22^. Additionally, preliminary genetic^20^ and metagenomic studies^23^ of these remains have been performed.

Here, we present the first analysis of mtDNA obtained from a large group of individuals living between the Oder and Vistula rivers during the IA. We identified mtDNA haplogroups for 40 individuals, and sequenced a complete mtDNA genome for 33 individuals. The performed analyses revealed a high genetic diversity of the studied group. Interestingly, women and men from Kowalewko had a significantly different genetic history. The collected data shed new light on the processes that shaped the specific, local genetic substructure of the human population in the South Baltic region during the IA.

## Results

### aDNA extraction, sequencing and preliminary characteristics

The studied group included people whose remains were excavated from the IA Wielbark culture cemetery, located in Kowalewko (Supplementary Fig. S1). There were two major reasons for choosing this particular cemetery. First, it is located in the central part of the region that spreads between the Oder and Vistula rivers. The genetic structure of the population living in this region during the IA and before has been poorly characterized. Second, the cemetery was used by the local population for 200 years. Therefore, it may be assumed that the obtained genetic profile would characterize reasonably well the population that lived there for a considerable period of time. The study included 60 individuals, whose burial sites have been thoroughly characterized archaeologically and anthropologically (detailed description in Supplementary Table S1). Because they represented the population living between the Oder and Vistula rivers during the IA, we called this group Kow-OVIA.

Radiocarbon dating (Supplementary Fig. S2 and Table S1) confirmed that the collected bone materials were from the first and second centuries (between AD 36 (+−30) and AD 181 (+−30)). aDNA was isolated from teeth, according to the procedure described by^24^ and^25^. In addition, for three individuals, aDNA was isolated twice, each time from a different tooth (internal control). In total, 63 aDNA samples were obtained. The NGS libraries were successfully obtained for 60 samples (for 57 individuals, including two libraries for three individuals from whom two samples were extracted). To estimate the amount of human DNA, all of the obtained libraries were subjected to shallow sequencing (the average coverage of mtDNA genome was 3.62x and ranged between 1 and 11x). The average amount of human DNA in the examined samples was 12% and ranged from 0.1 to 91.9% (Supplementary Table S2). In all of the samples, human DNA showed a typical aDNA damage pattern (C > T and G > A alteration most frequently occurring at the 5′ and 3′ ends of the reads) (Supplementary Table S3). The data obtained from the shallow sequencing enabled mtDNA haplogroup definition for 23 individuals. Importantly, the haplogroups obtained for the samples isolated from the same individuals were identical. Furthermore, for 27 individuals, their genetic sex was identified. The obtained results are summarized in Supplementary Table S4.

The shallow sequencing allowed for determination of a full sequence of mtDNA genomes for 13 individuals. In addition, 23 samples (22 individuals) with high amounts of endogenous DNA (over 10%) were subjected to deep sequencing (the average coverage was 163x and ranged between 6x and 1092x). As a result, a full sequence of mtDNA genomes was determined for 22 additional individuals (Supplementary Table S4). In all cases, mtDNA haplogroups were also established. For 20 individuals, mtDNA haplogroups were determined twice, i.e., based on shallow and deep sequencing data. Importantly, in all cases, particular individuals were classified into the same haplogroups. Next we assessed the contamination levels in all samples. To this end, we used the software contamMix. Mean value for authenticity estimates was 96%. Two individuals (PCA0018 and PCA0063) had lower than average authenticity probability, therefore were excluded from subsequent analyzes (Supplementary Table 1). In total, the full sequence of mtDNA was determined for 33 individuals and the haplogroup was established for 40 individuals (Supplementary Table S4). All identified haplogroups had been found earlier in the populations living in Central Europe from the EBA to the IA and belonged to 9 haplogroups (H, N, I, J, K, T, U, W, and X) (Table 1).

**Table 1:** Results of mtDNA haplogroup assignment.

Sample	Haplogroup confidence	Haplogroup	Sex assignment**
Sample_PCA0001	0,8	W	(M)/−
Sample_PCA0002	1	H28a1	(M)/−
Sample_PCA0003	1	H5a1	(F)/−
Sample_PCA0004	1	U3a1a1	(F)/−
Sample_PCA0005	—	U5b*	(M)/−
Sample_PCA0006	1	U5a1d1	(F)/−
Sample_PCA0007	1	W1	(F)/−
Sample_PCA0013	1	J1c3	(−)/−
Sample_PCA0014	—	—	(F)/−
Sample_PCA0015	1	H1f1a	(M)/M
Sample_PCA0016	—	—	(−)/−
Sample_PCA0017	—	—	(M)/−
Sample_PCA0018	0,5	HV18	(M)/M
Sample_PCA0019	—	—	(F)/−
Sample_PCA0020	—	—	(−)/−
Sample_PCA0021	—	—	(−)/−
Sample_PCA0022	—	—	(F)/−
Sample_PCA0023	—	—	(−)/−
Sample_PCA0024	—	—	(F)/−
Sample_PCA0025	—	—	(F)/−
Sample_PCA0026	0,5	T2b16	(F)/F
Sample_PCA0027	1	H1a	(M)/M
Sample_PCA0028	1	U3a1a	(F)/F
Sample_PCA0029	1	X2c1	(−)/−
Sample_PCA0030	1	H2a2b	(F)/−
Sample_PCA0031	1	K2a	(F)/F
Sample_PCA0032	0,5	T2n	(F)/F
Sample_PCA0033	—	—	(F)/−
Sample_PCA0034	1	K2a	(F)/F
Sample_PCA0035/65	0,5	J2b1a5	(M)/M
Sample_PCA0036	1	U5b1d1	(M)/−
Sample_PCA0037	0,5	T2e	(M)/M
Sample_PCA0038	1	H1e1a	(−)/−
Sample_PCA0039	—	—	(−)/−
Sample_PCA0040	1	I4a	(M)/M
Sample_PCA0041	0,8	N	(M)/−
Sample_PCA0042	—	—	(−)/−
Sample_PCA0043	—	—	(−)/−
Sample_PCA0044	1	H5a1	(F)/F
Sample_PCA0045	1	U5b1d	(F)/F
Sample_PCA0046a_b	1	U8a1a1b	(M)/M
Sample_PCA0047	1	H5a1	(F)/F
Sample_PCA0048	—	—	(−)/−
Sample_PCA0049	1	H1a	(F)/F
Sample_PCA0050	1	H1e1a	(M)/M
Sample_PCA0051_a_b	0,4	K2a	(−)/F
Sample_PCA0052	1	U5a1a1	(F)/F
Sample_PCA0053	1	K1b2a	(F)/F
Sample_PCA0054	1	U3a1a	(−)/F
Sample_PCA0055	—	—	(−)/−
Sample_PCA0056	1	T2b6a	(F)/F
Sample_PCA0057	1	J1c7a	(F)/−
Sample_PCA0058	—	—	(−)/−
Sample_PCA0059	1	K2a	(F)/F
Sample_PCA0060	1	U5b1d1	(M)/M
Sample_PCA0061	1	H1ak	(−)/−
Sample_PCA0062	1	U4a2	(M)/M
Sample_PCA0063	1	H2a5	(M)/M
Sample_PCA0064	—	—	(F)/−
Sample_PCA0066	1	H2a5	(M)/M

*Haplogroup assigned as in Juras, *et al*.^20^.

**Anthropological assignment given in (), genetic sex assignment given after/.

### Intrapopulation genetic diversity of Kow-OVIA

**Figure 1:**
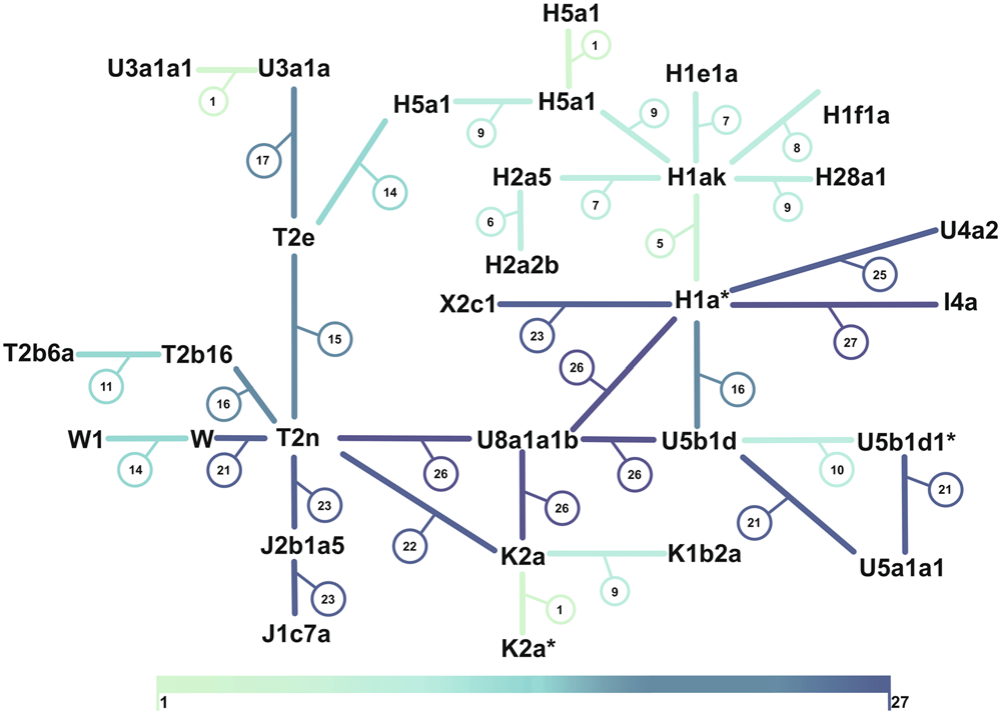
Minimal Spanning Network of 33 individuals from Kow-OVIA based on full mtDNA sequences. Each node corresponds to a haplotype determined for a unique mtDNA sequence. Numbers in circles show numbers of nucleotide differences between haplotypes. Edges are color-coded for the amount of nucleotide differences. The length of the edges is not informative. Stars mark haplotypes represented by two individuals with the identical mtDNA sequence.

The intrapopulation diversity of Kow-OVIA was analyzed using the full length mtDNA sequences obtained for 33 individuals (Supplementary Table S4). In a first step, we used a Minimum Spanning Network (MSN) method^26^ to identify and visualize differences between particular mitochondrial genomes (Fig. 1). The obtained graph was typical for the population with a high level of genetic diversity^27^. We identified 3 pairs of individuals with identical mtDNA sequences (PCA0059, PCA0031; PCA0049, PCA0027; PCA0060, PCA0036) and 4 pairs of individuals with only one different nucleotide in the mtDNA sequences (PCA0047, PCA0003; PCA0004, PCA0028; PCA0034, PCA0059; PCA0034, PCA0031). To exclude the possibility that the presence of two potentially maternally related individuals in relatively small dataset will affect the results of our consecutive analyses, we removed from the tested group three individuals - one from each pair with identical mtDNA sequences. For the comparison, the results obtained with the dataset containing potentially related individuals were shown in the Supplementary Figures S4–S6.

Next, we applied Arlequin ver. 3.5.1 to determine the levels of haplotype diversity (HD) based on two fragments of the HVS (hypervariable sequence) region (HVS-I between 16033 and 16365 np (nucleotide positions) and HVS-II between 73 and 340 np) (Supplementary Table S5), and nucleotide diversity (π) based on the fragment of the HVS-I region (between 16000 and 16410 np) (Supplementary Table S6). The HD level calculated for Kow-OVIA (1.0000 +/−0.0082) was similar to those currently observed for the contemporary, open European populations and higher (even when considering 95% confidence interval) than the HD obtained for the population considered isolated (Supplementary Table S5)^28^. The level of π determined for Kow-OVIA (0.007922 +/−0.004666) was only slightly lower than the average value obtained for the present-day European populations (0.009^29^) (Supplementary Table S6). Thus, the results of all three analyses, MSN, HD, and π, indicated that Kow-OVIA was not a genetically homogenous, isolated population.

### Kow-OVIA relationships with other populations from European space-time

#### mtDNA haplogroup frequency

**Figure 2:**
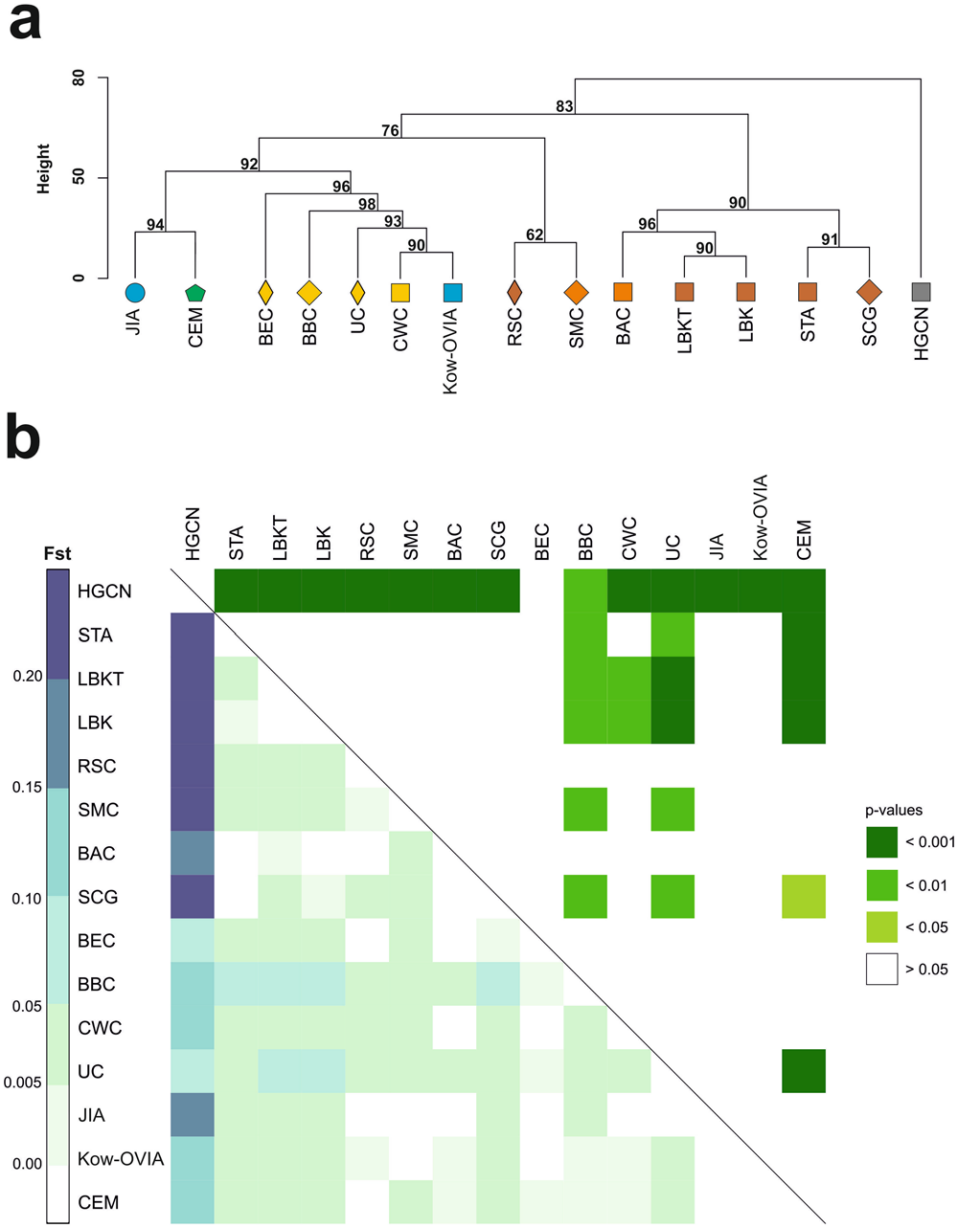
(**a**) Unsupervised hierarchical clustering with the Ward method and Euclidean distance on haplogroup frequencies for the CEPT populations. P-values of the clusters are given as the percent of reproduced clusters based on 10,000 bootstrap replicates. (**b**) Level plot of pairwise Fst values (below the diagonal) and corresponding p-values (above the diagonal) for the CEPT populations. P-values are based on 10,000 simulations and accounted for multiple comparisons with Benjamini-Hochberg corrections.

In the first step, we attempted to place Kow-OVIA in Central European space-time only. To this end, we compared mtDNA haplogroup frequencies in Kow-OVIA with other populations living in that region from the Mesolithic until the IA. The studied group, including Kow-OVIA and 13 other fossil populations (for details see Table 2 and Supplementary Table S7), was extended with the present-day Central Europe Metapopulation (CEM) (Supplementary Table S8). The obtained set of 15 populations called CEPT (Central European Population Transect) was subjected to unsupervised hierarchical clustering (Ward’s method with Euclidean distance) (Fig. 2a). The obtained results generally complied with known chronology of the formation of the genetic structure of the Central European population. The first to be separated from other populations were the HG. Next, we observed a divergence of the Early Neolithic (EN) populations [the Starčevo Culture (STA), Linearbandkeramik in Transdanubia (LBKT), Linearbandkeramik population from Central Europe (LBK), and Schöningen Group (SCG)] and the Middle Neolithic (MN) populations [the Baalberge Culture (BAC)] from the LN/EBA populations [the Bernburg Culture (BEC), CWC, BBC, and UC]. Then, the dendrogram supported the division into LN/EBA populations together with Kow-OVIA and post-EBA populations [Jutland Iron Age (JIA), CEM]. We noticed that Rössen Culture (RSC) and Salzmünde Culture (SMC) did not group with other EN/MN populations, but occupied a basal position with respect to the LN/EBA clade.

**Table 2:** Published reference ancient mtDNA data and populations abbreviations.

**Abbreviation**	**Population**	**n**
HGCN	Hunter Gatherers Central North Europe	23
HGSW	Hunter Gatherers South West Europe	13
HGE	Hunter Gatherers Eastern Europe	14
STA	Starčevo culture	44
LBKT	Linearbandkeramik culture in Transdanubia	39
LBK	Linearbandkeramik culture in Central Europe	102
RSC	Rössen culture	17
SCG	Schöningen group	33
BAC	Baalberge culture	19
SMC	Salzmünde culture	29
BEC	Bernburg culture	17
CWC	Corded Ware culture	44
BBC	Bell Beaker culture in Central Europe	35
UC	Unetice culture	94
PWC	Pitted Ware Culture	19
CAR	Cardial/Epicardial culture of the Iberian Penisula	18
NPO	Portuguese Neolithic population	17
NBQ	Neolithic population from Basque Country and Navarre	43
TRB	Funnel Beaker culture	10
TRE	Treilles culture	29
BAS	Bronze Age Siberia	11
BAK	Bronze Age Kazakhstan	8
RRBP	Gurgy ‘Les Noisats’ group	55
MIR	Iberian Chalcolithic El Mirador Burgos individuals	23
JIA	Jutland Iron Age	24
IIA	Iberian Iron Age population	27
SCY	Iron Age Scythian samples	16
SSP	Scytho-Siberian Pazyryk Culture	26
YAM	Yamnaya culture	41
Kow-OVIA	Oder Vistula Iron Age	40

For list of references see Supplementary Table S7.

**Figure 3:**
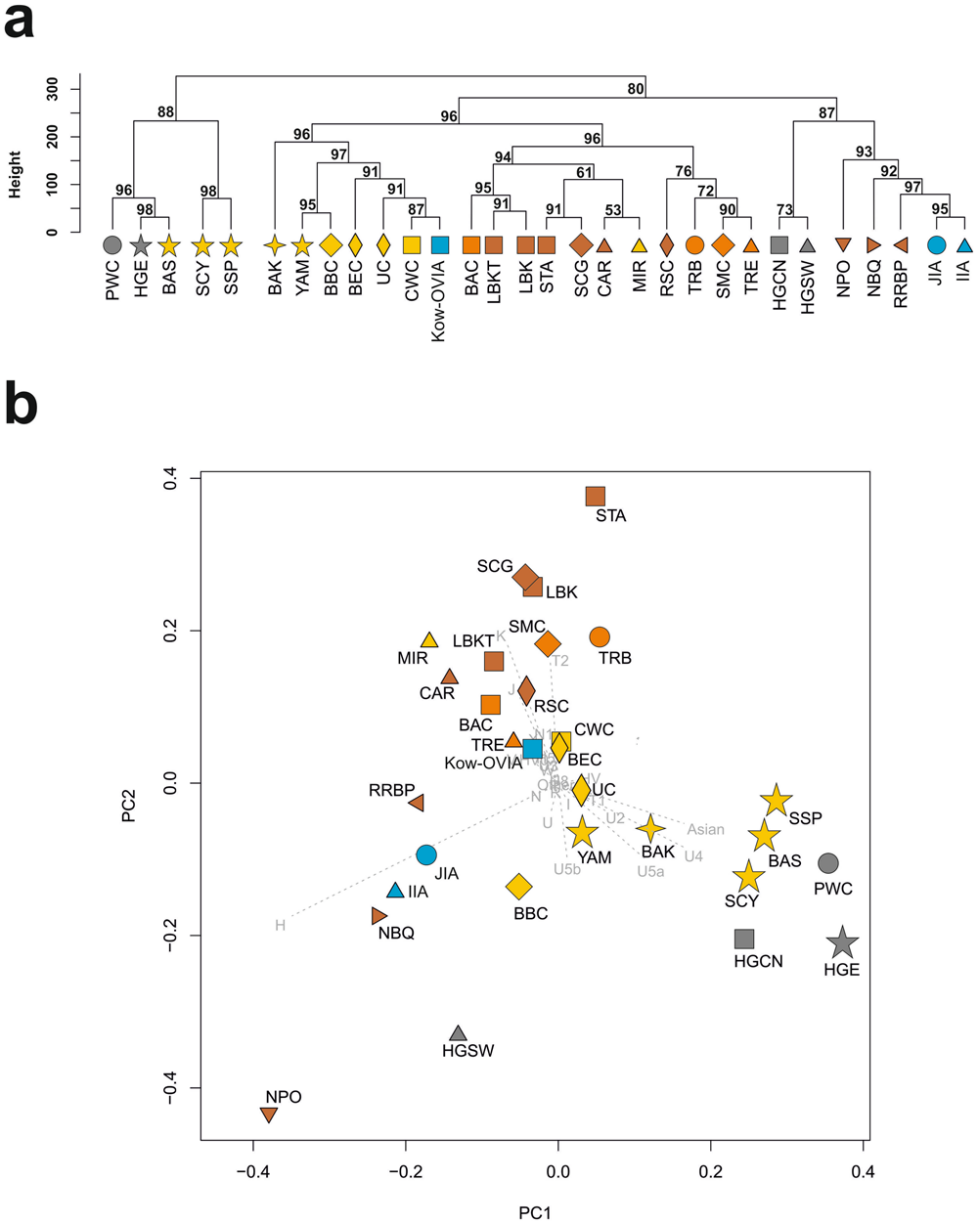
(**a**) Unsupervised hierarchical clustering with the Ward method and Euclidean distance on haplogroup frequencies for the EPT populations. P-values of the clusters are given as the percent of reproduced clusters based on 10,000 bootstrap replicates. (**b**) PCA on the haplogroup frequencies of EPT populations. Symbols indicate populations from Central Europe (squares and diamonds), Southern Scandinavia and Jutland Peninsula (circles), Iberian Peninsula (triangles), and East Europe/Asia (stars). Color shading of data points denotes Hunter-Gatherers (gray), Early Neolithic (brown), Middle Neolithic (orange), Late Neolithic/Early Bronze Age (yellow) and Iron Age (blue). The first two principal components of the PCA display 48.4% of the total genetic variation. Each haplogroup was superimposed as component loading vectors (gray dotted lines) proportionally to their contribution. Abbreviations: Central/North European Hunter-Gatherers (HGCN), Southwestern European Hunter-Gatherers (HGSW), East European Hunter-Gatherers (HGE), Starčevo Culture population (STA), Linearbandkeramik in Transdanubia (LBKT), Linearbandkeramik population from Central Europe (LBK), Rössen Culture (RSC), Schöningen Group (SCG), Baalberge Culture (BAC), Salzmünde Culture (SMC), Bernburg Culture (BEC), Corded Ware Culture (CWC), Bell Beaker Culture (BBC), Unetice Culture (UC), Pitted Ware culture (PWC), Funnel Beaker culture (TRB), Jutland Iron Age (JIA), Cardial/Epicardial culture of the Iberian Peninsula (CAR), Portuguese Neolithic population (NPO), Neolithic population from Basque Country and Navarre (NBQ), Iberian Chalcolithic El Mirador Cave individuals (MIR), individuals from Iberian Iron Age period (IIA), Treilles Culture (TRE), Gurgy ‘Les Noisats’ group (RRBP), Bronze Age Kurgan samples from South Siberia (BAS), Bronze Age Kazakhstan (BAK), Yamnaya (YAM), Iron Age Scythian (SCY), Scytho-Siberian Pazyryk Culture (SSP), Kowalewko Oder and Vistula Iron Age (Kow-OVIA).

To place the Kow-OVIA in a context broader than the Central European one, we removed CEM from the studied group and extended it with 16 populations that lived in other regions of Europe from the Mesolithic to the IA (Supplementary Table S7). The obtained set of 30 populations (European Population Transect, abbreviated EPT) as earlier was subjected to hierarchical clustering and additionally to PCA (Fig. 3a and b, Supplementary Table S9). Both analyses could distinguish one major group composed of EN/MN populations, however, excluding the Neolithic populations of the Iberian Peninsula (Neolithic Portugal (NPO) and Neolithic Basque Country (NBQ)). Within NPO and NBQ, the mtDNA haplogroup H was observed with a higher frequency than in the other Neolithic populations. LN populations were more scattered and their distribution seemed to be associated with the geographical location. For the populations inhabiting east peripheries of Europe [YAM, Bronze Age Kurgan samples from South Siberia (BAS), Iron Age Scythian (SCY) and Scytho-Siberian Pazyryk Culture (SSP)], the most characteristic were both the mtDNA haplogroups typical for Asia (mainly C, D) and a high prevalence of haplogroups U5a and a lack of U5b (except the population of Bronze Age Kazakhstan (BAK)) for almost the entire analyzed period of time. Interestingly, the closely located in time and space Kow-OVIA and JIA occupied the opposite side of the PCA diagram (Fig. 3b) than the preceding LN/EBA populations (the BEC, CWC, and UC). Such a placement resulted from the higher prevalence of the haplogroup H in Kow-OVIA and JIA compared to the BEC, CWC and UC. Alternatively, Kow-OVIA and JIA showed considerable differences in the prevalence of haplogroups typical for LN/EBA (I, R, T1, U2). They were less frequently observed in Kow-OVIA (2.7%) than in JIA (16.6%). As expected, the HG did not form a single group and differed from the other populations by a considerable portion of haplogroup U and a general lack of haplogroups associated with the “Neolithic package”.

Our analyses also revealed very interesting and unreported earlier changes in the frequency of haplogroups U5a/U5b in the Central European populations (Supplementary Table S8). Initially, during the LN/EBA, the haplogroup U5a, characteristic for the populations from eastern glacial refugia, dominated. Then, during the IA, haplogroup U5b, typical for the populations from the western glacial refugia, was more frequent. At present, haplogroup U5a is again more often found in the CEM.

We also placed Kow-OVIA on a map showing the matrilineal genetic structure of the present-day human population. To this end, we performed a PCA of haplogroup frequencies of Kow-OVIA and 73 extant worldwide populations (Supplementary Fig. S3a and Table S10, Supplementary Material Text). Kow-OVIA was located within a range of European genetic diversity, in close proximity to the present Scandinavian populations (Norwegian and Swedish).

#### Genetic distances

The relationships between Kow-OVIA and the earlier and later European populations were also determined by measuring genetic distances among them. In the first step, we focused on populations from Central Europe only (from the CEPT group). Our analysis involved a fragment of mtDNA HVS-I sequence (the region between 16064 and 16400 np) (Fig. 2b and Supplementary Table S11). The established fixation indexes (Fst) showed that Kow-OVIA was most closely related to the BEC (Fst = −0.01119, p = 0.74513) and JIA (Fst = −0.01054, p = 0.81012). CEM showed the lowest genetic distances to JIA (Fst = −0.00512, p = 0.75990), and Kow-OVIA (Fst = −0.00349, p = 0.74513).

**Figure 4:**
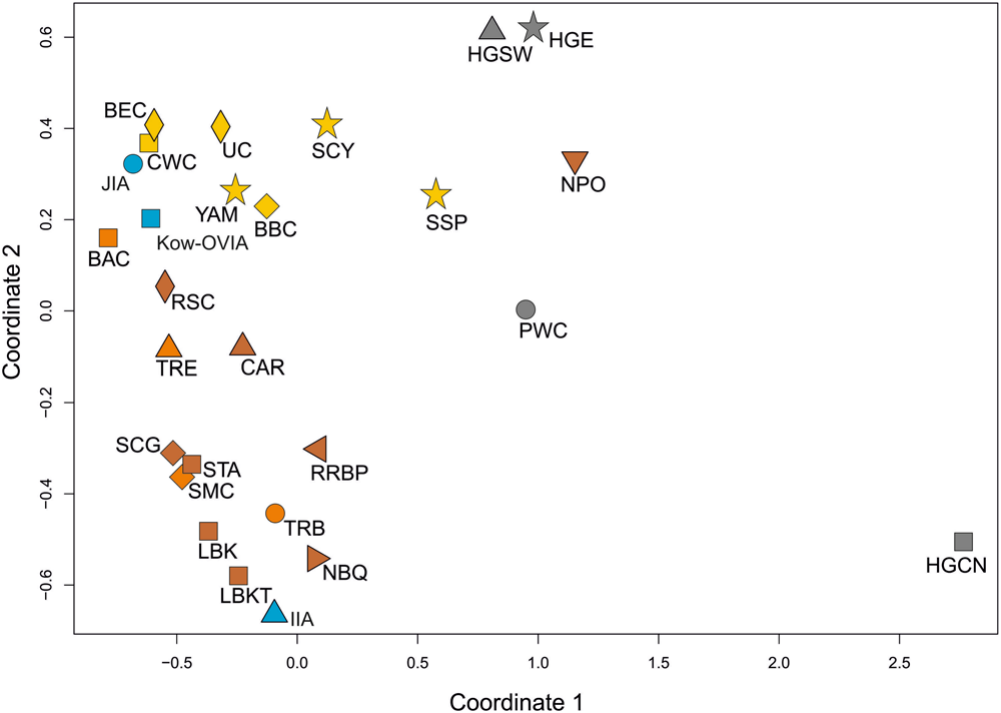
MDS plot of Slatkin’s Fst values for EPT populations. Fst values were obtained for mtDNA HVS-I region (16064–16400 np). Symbols and color shading as in Fig. 2.

In the second step, we determined the genetic distances between Kow-OVIA and populations living in the whole of Europe from the Mesolithic age to the IA. To this end, we selected 26 populations from the EPT group for which sequences of the mtDNA HVS-I region between 16064 and 16400 np (Supplementary Table S12) were known for a considerable number of individuals. The calculated genetic distances (Supplementary Table S12) again showed that Kow-OVIA was closest to the BEC (Fst = −0.01097, p = 0.72712) and then to the JIA (Fst = −0.01054, p = 0.80627). The results were visualized on a multidimensional scaling (MDS) plot (Fig. 4). As in the analysis of the haplogroup frequencies, we observed that although separated by a short distance, Kow-OVIA and JIA were both differently located regarding particular LN/EBA populations (the BBC, CWC, and UC). The distance between the Kow-OVIA and BBC (Fst = 0.00342; p = 0.36707) is five times smaller than for the JIA - BBC pair (Fst = 0.02426; p = 0.07196). Simultaneously, the relationship is opposite when one compares the Kow-OVIA and JIA with the CWC and UC (Fig. 4 and Supplementary Table S12). In addition, the performed analysis showed a previously unnoticed small genetic distance between the YAM and BBC (Fst = −0.00463, p = 0.58485), and the YAM and CWC (Fst = −0.00157, p = 0.50459).

### Turning points in the formation of the Central European genetic structure

To verify the existing hypothesis describing the formation of the genetic structure of the Central European population in the period between the EN and IA, we used the analysis of molecular variance (AMOVA). In the first step, we determined an optimal division of the populations from CEPT excluding the HG and CEM. The division was considered to be optimal if intragroup variability (Fsc) was minimal and intergroup variability (Fct) maximal. In our analysis, we took advantage of earlier observations made by^2^ and^10^ who demonstrated relatively high genetic homogeneity of the EN/MN population (STA, LBKT, LBK, RSC, SCG, BAC, SMC). Accordingly, we allocated all EN/MN populations to one group and analyzed 18 different combinations of population grouping (Supplementary Table S13). We found that Fsc and Fct were optimal if the studied populations were divided into three groups: EN/MN (the STA, LBKT, LBK, RSC, SCG, BAC, and SMC), LN/EBA/IA (the BEC, UC, and JIA), and LN/IA (the CWC, BBC, and Kow-OVIA) (Fsc 0.00274, p = 0.24891 + −0.00436; Fct 0.02553, p = 0.00000 + −0.00000). The obtained results suggest that the JIA was closely related to the North-Central Europe populations, whereas Kow-OVIA was related to the populations of both the Eastern and Western regions of Europe.

Next, we performed an analogous analysis but the studied group was extended by CEM (CEPT with excluded HG populations). Once again, we assumed that the populations of the EN/MN (STA, LBKT, LBK, RSC, SCG, BAC, and SMC) are a homogenous group, and we analyzed 33 combinations of the other fossil populations and CEM (Supplementary Table S14). The combination of 3 groups (the STA, LBKT, LBK, RSC, SCG, BAC, and SMC; the BEC, and UC); and the CWC, BBC, Kow-OVIA, JIA, and CEM) revealed better optimization of Fsc (0.00242, p = 0.29426 + −0.00396) and Fct (0.02166, p = 0.00050 + −0.00022) than for the two-group correlation (STA, LBKT, LBK, RSC, SCG, BAC, and SMC; and the BEC, CWC, BBC, UC, Kow-OVIA, JIA, and CEM) (Fsc = 0.00593, p = 0.03545 + −0.00175; Fct = 0.02253, p = 0.00050 + −0.00022). We also found that better optimization of the Fsc and Fct parameters was observed for the combinations where Kow-OVIA and JIA formed a group with the CEM, contrary to the combinations where the CEM was grouped with the LN/EBA populations (Supplementary Table S14). The results of the above analyses suggested that the contribution of the particular populations to the genetic structure of present-day Europe is not fully consistent with the current chronology of which populations inhabited Central Europe. The JIA, Kow-OVIA, BBC and CWC contributed more significantly to the genetic structure of present-day Europe than the BEC and UC.

### Analysis of mtDNA lineages

To obtain more evidence to verify the above observations, we determined which populations gave rise to haplotypes found in Kow-OVIA (Supplementary Table S15). The same analysis of shared ancestral haplotypes^10^ was performed for JIA (Supplementary Table S15). We observed a higher incidence of the HG mtDNA lineages in Kow-OVIA (9.68%) than in JIA (0%) and CEM (2%). Simultaneously, Kow-OVIA showed a lower incidence of the lineages originating from the LN/EBA (6.45%) compared to the JIA (29.16%) and CEM (13.62%).

Moreover, we performed a traditional analysis of shared haplotypes^30^ (Supplementary Table S16), which demonstrated that approximately 29.4% of the haplotypes inherited by the Kow-OVIA were common for LN/EBA populations (BBC, CWC, and UC). These haplotypes came from haplogroups H and U5a. The haplotype that belonged to U5a, according to current knowledge, first occurred in the LN/EBA, whereas the H haplotypes showed continuation from the EEF (Starčevo). The JIA inherited only approximately 23% of the haplotypes shared by LN/EBA populations (BBC, CWC, UC). Approximately 41% of Kow-OVIA and 19% of JIA haplotypes were not found in any LN/EBA population although the latter preceded them.

### Sex-linked factors affecting the genetic structure of Kow-OVIA

Finally, we attempted to determine whether the processes shaping the genetic structure of the Kow-OVIA population were biased by sex. To this end, based on anthropological and genetic sex assignment (Table 1), we divided the studied group into female and male subgroups – Kow-OVIA-F (17 individuals) and Kow-OVIA-M (14 individuals), respectively. Both subgroups were subjected to the analogical analyses as previously performed for the entire Kow-OVIA.

**Figure 5:**
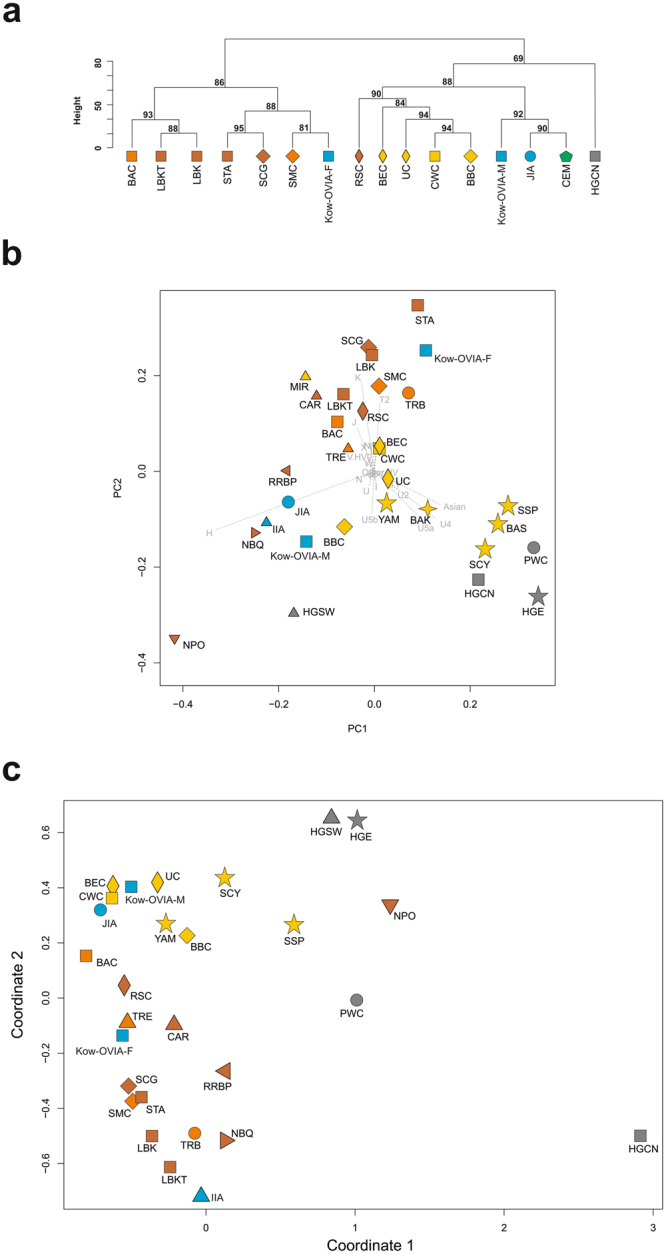
Analysis of haplogroup frequencies in the EPT populations, with Kow-OVIA female (Kow-OVIA-F) and male (Kow-OVIA-M) subgroups: (**a**) unsupervised hierarchical clustering with the Ward method and Euclidean distance, P-values of the clusters are given as the percent of reproduced clusters based on 10,000 bootstrap replicates; (**b**) PCA, each haplogroup was superimposed as component loading vectors (gray dotted lines) proportionally to their contribution; (**c**) MDS plot of Slatkin’s Fst values, obtained for mtDNA HVS-I region (np 16064–16400). Symbols and color shading as in Fig. 2.

We found that the Kow-OVIA-F had a higher π value than the Kow-OVIA-M: 0.009185+/−0.005470 and 0.006987 0.004413, respectively (Supplementary Table S6). However, the difference was not statistically significant. Unsupervised hierarchical clustering involving CEPT showed that Kow-OVIA-M, similar to Kow-OVIA, grouped with the JIA and CEM whereas Kow-OVIA-F was placed in the diagram among the EN and MN populations (Fig. 5a and Supplementary Table S8).

PCA analysis of Kow-OVIA-F, Kow-OVIA-M and EPT populations (Fig. 5b and Supplementary Table S9) once again showed that Kow-OVIA-M was located similarly to Kow-OVIA, i.e., near the BBC and JIA due to considerable sharing of haplogroups H and U5b. However, the Kow-OVIA-F was grouped with the EN and MN populations, which was mainly a result of a high prevalence of the haplogroup K. The existence of genetic differences between females and males was also supported by the Fisher’s test on the haplogroup frequencies (p = 0.013).

The PCA of haplogroup frequencies of Kow-OVIA-F/M and 73 extant worldwide populations again revealed high genetic differences between Kow-OVIA-F and Kow-OVIA-M (Supplementary Fig. S3b and Table S10). Although for both, the closest are contemporary European populations (PC1 and PC2), Kow-OVIA-F and Kow-OVIA-M occupy opposite sites on PC3 (in relation to the contemporary European populations).

Analysis of genetic distances between the Kow-OVIA-F/M and particular populations of the CEPT (Supplementary Table S17) also showed significant differences in the genetic history of the mtDNA lineages of male and female individuals. Kowalewko males showed the smallest genetic distance to the JIA (Fst = −0.01249, p = 0.74854), whereas females had smallest genetic distance to the BEC (Fst = −0.01598, p = 0.72236, respectively). The genetic distances between Kow-OVIA-F/M and BBC/CWC/UC/CEM were asymmetrical. The Kow-OVIA-M, as well as JIA, showed lower distances to CWC, UC and CEM than Kow-OVIA-F. Kow-OVIA-M was also located closer to BBC than Kow-OVIA-F (Fst = 0.00900, p = 0.41444, Fst = 0.01372, p = 0.39184, respectively). In this case, the distance between JIA and BBC (Fst = 0.02429, p = 0.13348) was twice as large as the distance between Kow-OVIA-F and BBC and much longer than between Kow-OVIA-M and BBC. The comparison of the Kow-OVIA-M and Kow-OVIA-F with older populations showed that: (i) the subgroup of women was genetically closer to the EN/MN populations (not including the BAC) than the male individuals; and (ii) the male subgroup was closer to the LN populations. We also verified the locations of Kow-OVIA-F and Kow-OVIA-M within the EPT set (Supplementary Table S18). The MDS analysis (Fig. 5c) demonstrated that the male individuals had smaller genetic distances to HG populations (HGCN, HGSW, HGE, and PWC) than did women and were closer to YAM than women (Fst = −0.00171, p = 0.48936; Fst = 0.01696, p = 0.21600, respectively). The analysis of shared haplotypes supported the described above tendencies - women from Kowalewko grouped with the EN and MN populations and were less connected with the BBC than men (Supplementary Table S19).

## Discussion

Despite tremendous progress that has recently been made in archaeogenomic studies of European populations, our knowledge of the changes that occurred in the genetic structure of the peoples inhabiting Central Europe between the LN/EBA and Middle Ages is still very limited. One major problem is the scarcity of genetic data describing individuals who occupied the region east of the Oder river. To fill this gap, we performed an analysis of the mitochondrial genomes of a large group of people who lived in the area between the Oder and Vistula rivers (present-day western Poland) during the IA (the 1^st^ and 2^nd^ centuries AD). The studied group initially comprised 60 individuals; for 40 individuals, we assigned the mtDNA haplogroup, and for 33 of them, we determined the sequence of the entire mitochondrial genome. This dataset is particularly important to understand demographic processes that occurred in Central Europe during the IA and in the context of migrations between the 3^rd^ and 6^th^ centuries AD that are believed to have shaped the genetic landscape of contemporary Europe^31^.

According to common knowledge, the region between the Oder and Vistula rivers 2 tya was densely wooded with single isolated human settlements scattered in the forest. It is thought that inhabitants of these settlements had very limited contact not only with the so-called “civilized world” but also with neighboring populations. Interestingly, the picture emerging from the latest archaeological studies of burials and artifacts seems to be different. Recently, it has been suggested that the settlement in Greater Poland was compact and regular, with the appearance and disappearance of the sites occupied by the arriving groups interacting with the surrounding populations^32^ and references therein. Our results strongly support the hypothesis presented by^32^. The analyses of intrapopulation variability revealed that the studied group was not an isolated population. The calculated HD and π levels fell within the range of values characteristic for contemporary open European populations.

Based on our results of mtDNA haplogroup frequency analysis, we were able to update a phylogenetic tree describing the history of *H*. *sapiens* in Central Europe (see Fig. 2a). Compared with the previous phylogenetic tree^2^, the new tree was expanded with the IA populations inhabiting the region between Oder and Vistula rivers and the region of Jutland. In the earlier constructed tree, CEM was closest to BBC despite the latter coexisting in Central Europe with the CWC (LN) and followed by the UC (EBA). In the new tree, Kow-OVIA was clustered with the LN/EBA populations. The present-day European population (CEM) was clustered with the JIA that was contemporary to Kow-OVIA and inhabited Jutland.

Based on the results presented above one can assume that there are some discrepancies between the hierarchical clustering (Fig. 3a) and PCA (Fig. 3b) of haplogroup frequencies in the EPT (for example, the placement of the Kow-OVIA in relation to the Treilles Culture population (TRE)). These differences indicate that in contrast to dendrogram, the PCA plot (capturing only first two principal components) did not show the full spectrum of EPT variability. We explored this issue by plotting additional principal components (Supplementary Figure S7a and b). It demonstrated that their contribution to the explanation of the genetic variability of EPT populations was non-trivial.

Analysis of genetic distances (see Fig. 2b) showed that both JIA and Kow-OVIA, are the closest to the CEM. However, it should be mentioned that many of the resulting genetic proximities did not reach statistical significance at the alpha level 0.05 (mainly due to the multiple comparisons), thus they should be interpreted with caution (Supplementary Table S11). Higher prevalence of the mtDNA haplogroup H in Kow-OVIA and JIA (its high level is also characteristic for the BBC) than in the preceding CWC and UC supports the hypothesis assuming significant demographic changes in Central Europe after the LN/EBA period^2^. This hypothesis is additionally strengthened by the results of AMOVA analysis indicating that there is some inconsistency between genetic distances and the chronology of the appearance of the studied populations in Central Europe, i.e., the older populations (BBC, CWC) contributed more to the genetic structure of CEM than the younger ones (UC).

Changes in the occurrence of mtDNA haplogroups U5a/U5b in Central Europe are also worth noting. At LN and EBA, the prevailing haplogroup was U5a for BBC/CWC/UC. Next, there was a dominance of U5b for the Kow-OVIA/JIA during IA and now U5a is again more popular (CEM). The first alteration in the U5a/U5b prevalence between the LN/EBA and the IA supports the hypothesis of demographic changes right after the LN, proposed by^2^. The second conversion indicated by our results suggests another crucial demographic event that should occur between the IA and present.

On the basis of the above observations, one may assume that in the IA, specific genetic substructures were formed in Central Europe. Because the demographic history of fossil populations often has a local character^33,34^, it is worth considering the range of the observed changes. These considerations should also take into account the hypothesis on the migrations that most likely occurred between the 3^rd^ and 6^th^ century AD. In this context, it seems necessary to compare Kow-OVIA and JIA with other populations from the IA, in particular those located east of Vistula, and with the populations that inhabited this region during the Middle Ages.

The process of demographic change in Central and Northern Europe after the LN/EBA appears to have a complex nature. Genetic proximity of the Kow-OVIA and JIA is consistent with the Kow-OVIA’s affiliation to the Wielbark archaeological culture, strictly connected with Baltic regions^32^. However, despite close genetic distance between Kow-OVIA and the JIA, PCA placed them asymmetrically in relation to other ancient populations (see Fig. 3b). According to MDS (Fig. 4), JIA was close to the North-East European populations (CWC, BEC, UC) whereas the relationships of Kow-OVIA with other populations (earlier and contemporary to Kow-OVIA) were not so obvious. Interestingly, a small genetic distance between the JIA and UC was correlated with a high prevalence of the mtDNA haplogroup I in both populations. This result is consistent with earlier hypotheses suggesting that the genetic structure of a contemporary Danish population was formed not later than in the IA^35^. Unfortunately, our knowledge of haplogroup I prevalence in the Nordic Bronze Age is still scarce because of the small number of analyzed individuals; thus, it cannot be unambiguously stated that the observed proximity of the JIA and UC was, indeed, a result of the demographic changes after the LN/EBA. However, the above conclusion postulating a close connection between JIA and UC is also supported by the result of shared haplotype analysis. We found that 25% of the ancestral haplotypes found in the JIA were first reported in the UC and were not common in any earlier population. To better understand this phenomenon, the analysis of Y chromosome haplogroups is required, as the Nordic Bronze Age is characterized by the occurrence of the Y-haplogroups I and I1^18^, whereas the UC is mainly characterized by a higher prevalence of I2^13^. More detailed Y chromosome analyses involving a larger number of individuals would also shed more light on the process that resulted in a high prevalence of the mtDNA H haplogroup in the JIA, which is another signal of post-LN demographic changes^2^. In the case of Kow-OVIA, its genetic root in multiple European populations is evidenced by the fact that 55% of the ancestral haplotypes that were identified in this group were common to populations of the LN/EBA period (the CWC, BBC, and UC). For the JIA, the same origin is observed for only 19% of ancestral haplotypes.

Finally, we found that the genetic structures of female and male subpopulations of Kow-OVIA were significantly different. This fact cannot be explicitly determined based on the results of individual analyses; however, it is quite evident if one considers the whole set of data presented here including the Fisher test on haplogroup frequencies. The analyses of both mtDNA haplogroups and genetic distances indicated that women from Kowalewko were related closer to the EN/MN populations, and the men were closer to the CWC and UC. This observation may explain why the genetic relationships of Kow-OVIA with other ancient European populations were more complex and more difficult to define as it was in the case of JIA. In analyzing Kow-OVIA, we observed multiple overlapping effects of two subpopulations with different genetic affinities. One would speculate that the genetic profile of Kow-OVIA-F resulted from exogamy that was described for the CWC population^36^. This is, however, not the case. We found that the genetic differences between women and men were maintained for the entire observation period, i.e., for 200 years (approximately 8 generations). Such a composition of the genetic structure of Kow-OVIA could exist only if at least one subgroup (Kow-OVIA-F or -M) was periodically exchanged. It would further mean that Kowalewko played some specific roles in that region. According to the recent archaeological studies, the colonization pattern in IA Greater Poland could be linked with the existence of a centralized organization system^32^. Kowalewko could have been one of the important elements of this system. For example, it could have functioned as a garrison for the population closely associated with the JIA, such that warriors stayed in the garrison for only a few years and were then replaced by others. Other scenarios are also possible; however, verification of any hypothesis requires more detailed studies.

## Materials and Methods

### aDNA extraction and library preparation

For the purposes of this study, 63 samples (teeth) were obtained from 60 individuals from the Kowalewko archaeological site (Supplementary Table S1). After being transported to a clean aDNA lab, the teeth were cleaned with 5% NaOCl and rinsed with sterile water, followed by UV irradiation (254 nm) for 2 hours per site. The roots of the teeth were drilled using Dremel® drill bits. Bone powder (approximately 250 mg) was digested with proteinase K and DNA-containing extract was purified using a silica-based method following^24^ and^37^. Genomic libraries were prepared following a protocol from^38^ omitting the initial sonication step, due to the natural fragmentation of aDNA. Six separate PCR reactions were set up for each library. PCR amplifications were performed in 25 µl with 3 µl of the DNA library template, 12.5 µl of 1x AmpliTaq Gold® 360 Master Mix (Life Technologies, California), 0.5 µl of indexing primer (10 µM) and 0.5 µl of PCR primer IS4 (10 µM) (Günther *et al*. 2015). The PCR profile was as follows: initial denaturation (94 °C, 12 min), 12–16 cycles of 94 °C (30 s), 60 °C (30 s), 72 °C (45 s) and final extension (72 °C, 10 min). PCR reactions for the same library were pooled and purified with AMPure® XP beads (Agencourt-Beckman Coulter) following^39^. Quality and size distribution of the libraries were verified with a High Sensitivity DNA kit and 2100 Bioanalyzer system (Agilent) while DNA concentration was determined with a Qubit fluorimeter and Qubit dsDNA HS Assay Kit (ThermoFisher Scientific), according to the manufacturers’ protocols.

### Next generation sequencing of aDNA libraries

For shallow sequencing, Genome Analyzer GAIIx (Illumina) and TruSeq SBS Kits v5-GA (Illumina) were applied. Seven to nine libraries were pooled per lane. On average, 4.75 mln 75 bp-long reads were collected per library. The libraries with the highest human DNA content were sent for deep sequencing to Macrogen Inc., Korea, with the use of HiSeq4000 (Illumina). On average, 87 mln 101 bp-long reads were collected per library on the HiSeq4000 (Illumina) (Supplementary Table S2).

### Filtering, mapping and variant calling

Raw sequencing data (fastq files) were filtered with AdapterRemoval^40^ by trimming missing nucleotides from both ends with the threshold of a minimum quality of 30 and a minimum length of 25 nucleotides. Filtered reads were aligned with BWA ver. 0.7.10^41^ to the rCRS mitochondrial, and GRCH 37 reference genomes, with seed blocked for higher sensitivity and other parameters set as default, as suggested in^42^. Following the alignment, duplicate reads were removed with picard-tools ver. 1.117 MarkDuplicates. Read depth and coverage were assessed with samtools ver. 1.2^43^ and bedtools ver. 1.2^44^. Consensus fasta sequences for haplogroup prediction and sequence analyses were generated with FreeBayes ver. 1.0.2–33-gdbb6160^45^ calling the base supported by the 3/5 majority of reads (Supplementary Table S4).

### Human DNA damage patterns

To examine data authenticity, we used mapDamage 2.0^46^ to estimate human DNA damage parameters typical for aDNA for each sample: (i) λ, the fraction of nucleotides positioned in the single-stranded DNA overhang context; (ii) δs, C→T substitution rate in the single-stranded overhang context; and (iii) δd, C→T substitution rate in the double-stranded DNA context (Supplementary Table S3).

### Contamination assessment

To estimate the level of contamination with contemporary human DNA or with other human aDNA we used the software contamMix to compare the alignment rates between estimated sample’s consensus mtDNA sequence and 311 potential contaminant mtDNA sequences.

### Analysis of intrapopulation genetic diversity

To assess intrapopulation diversity of Kow-OVIA, we used the MSN method on 33 full mtDNA sequences. HD was analyzed with Arlequin ver. 3.5.1^47^ on two fragments of mtDNA HVS region (HVS-I, 16033–16365 np, and HVS-II 73–340 np) (Supplementary Table S5). π was calculated for the fragment of mtDNA HVS-I region (16000–16410 np) (Supplementary Table S6).

### mtDNA haplogroup frequency analyses

Haplogroups were predicted with HaploFind^48^ with respect to Phylotree build 17 (http://www.phylotree.org/)^49^. Only samples with a haplogroup score ≥ 0.8 and average coverage ≥ 3 were used in downstream analyses. To assess temporal mtDNA haplogroup frequency changes from Mesolithic to the present day, we performed Ward clustering with Euclidean distance on 23 haplogroups (H, H5, HV, HV0, V, I, J, K, N, N1a, R, T1, T2, U, U2, U3, U4, U5a, U5b, U8, W, X and others). We used a set of 40 samples from this study and 13 populations from Central/North Europe (Supplementary Table S7), and a generated Central European Metapopulation (CEM) composed of 500 random individuals sampled from the extant populations of Poland, Czech Republic, Germany and Austria, as in^2^ (Supplementary Table S8).

To compare mtDNA variability of Kow-OVIA in a broader geographical context, we also applied unsupervised hierarchical clustering with the Ward method and Euclidean distance, and PCA on haplogroup frequencies of Kow-OVIA and published ancient mtDNA data from Mesolithic, Neolithic, Bronze Age and Iron Age populations from across Europe and West Asia (Supplementary Table S7). Haplogroups were divided into 25 groups present in ancient individuals (Asian [A, C, D, F, G, Z], N, N1a, I, J, W, X, R, HV, V/HV0, H, H5, T, T1, T2, J, U, U2, U3, U4, U5a, U5b, U8, K and others) (Supplementary Table S9).

To elucidate affinities of our samples in relation to present day populations, we performed a PCA analysis based on 23 haplogroup frequencies (Asia [A, C, D, F, G, Z], Africa [L], N1a, I, I1, W, X, HV, V/HV0, H, H5, T1, T2, J, U, U2, U3, U4, U5a, U5b, U8, K and others), with public data from 73 extant populations (Supplementary Table S10).

Cluster significance was tested by performing 10,000 permutations with the pvclust package in R ver. 3.3.0. PCA was conducted with prcomp of the vegan package in R ver. 3.3.0 (http://R-project.org).

### Genetic distance analyses

For sequence based analyses, the longest mtDNA HVS-I fragment present in the biggest fraction of published samples was selected (16064–16400 np). Additionally, for newly reported samples from this study, no missing nucleotides were allowed in the selected HVS-I range, and at least 3x coverage was expected for 95% of the nucleotides. To examine genetic affinities on the sequence level, we calculated genetic distances (Fst)^50^ between two sample sets: CEPT (Supplementary Table S11 and S17) and EPT (Supplementary Table S12 and S18), including only those individuals for which the 16064–16400 fragment of mtDNA HVS-I was present.

Pairwise and Slatkin’s Fst^51^ values were calculated in Arlequin ver. 3.5.1 for both datasets separately, with associated substitution model and gamma values selected with jModel test 0.1 AIC and BIC^52^. P values were calculated by performing 10,000 permutations. Genetic distances were visualized on an MDS plot with metaMDS function from the vegan package in R ver. 3.3.0.

### Analysis of genetic structure

To examine if genetic affinities between particular populations from CEPT were a result of shared genetic structure, we conducted AMOVA analysis in 18 combinations of CEPT populations (excluding HG and CEM) (Supplementary Table S13). Next, we tested the genetic contributions of ancient populations to the extant mtDNA variability by analyzing AMOVA results from 33 combinations of CEPT populations (excluding HG) (Supplementary Table S14). Statistical significance was obtained by performing 10,000 permutation tests.

### mtDNA lineage analyses

To further test genetic affinities and account for temporal succession of archaeological cultures in Central Europe, we conducted an analysis of shared ancestral haplotypes as described in^10^ in CEPT (Supplementary Table S15) and classical shared haplotype analysis^30^ in LN/EBA and IA populations (Supplementary Table S16 and S19).

For details, see the Supplementary Materials.

### Availability of data and material

The data supporting the results of this article are available at SRA, BioProject PRJNA354503.

## Electronic supplementary material

Supplementary dataset

Supplementary information
