# Supplementary material for: A mosaic genetic structure of the human population living in the South Baltic region during the Iron Age
Source: Sci Rep. 2018 Feb 6;8:2455. doi: 10.1038/s41598-018-20705-6 (PMC5802798; doi:10.1038/s41598-018-20705-6)
Supplement: Supplementary file 2 — Supplementary information [file 41598_2018_20705_MOESM2_ESM.doc]

Supplementary Materials for:

**A mosaic genetic structure of the human population living in the South Baltic region during the Iron Age**

Ireneusz Stolarek^1^, Anna Juras^2^, Luiza Handschuh^1^, Małgorzata Marcinkowska-Swojak^1^, Anna Philips^1^, Michał Zeńczak^1^, Artur Dębski^3^, Hanna Kóčka-Krenz^3^, Janusz Piontek^2^, Piotr Kozłowski^1^, Marek Figlerowicz^1,4^*

^1^ Institute of Bioorganic Chemistry, Polish Academy of Sciences, Poznan, Poland

^2^ Institute of Anthropology, Faculty of Biology, Adam Mickiewicz University, Poznan, Poland

^3^ Institute of Archaeology, Collegium Historicum, Adam Mickiewicz University, Poznan, Poland

^4^ Institute of Computing sciences, Poznan University of Technology, Poznan, Poland

^*^ Correspondence and requests for materials should be addressed to:

Prof. Marek Figlerowicz

Institute of Bioorganic Chemistry

Polish Academy of Sciences

Noskowskiego 12/14

61-704 Poznan, Poland

email: marekf@ibch.poznan.pl

**Materials and Methods**

**Archaeological sites and dating**

Kowalewko is a village in Wielkopolskie vojevodship, close to Poznan, in the middle reaches of the Samica Kierska river. Biritual Roman Age cemetery (site 12), dated from the mid-1st to the beginning of 3rd century AD, is located in the featureless arable fields at the South and West of the village. It is one of the most significant and valuable burial sites identified with the Wielbark Culture in Poland. The "untouched" nature of the cemetery and favorable burial environment enabled the survival of bones and artefacts in good condition. As a result of excavations, conducted in 1995-1998, 496 graves (including cremation burials and inhumation graves) were discovered, including 5 under burial mounds (supplementary fig. S1). The graves, representing the plenty of burial forms typical for the Wielbark Culture, were classified to the subphases B1b to C1a, based on the type of the burial rite, the body position, the found goods and the grave constructions. The furnishing of the graves includes dress accessories, ornaments and everyday objects such as pottery vessels, combs or spindle whorls, but specialist tools and weapons were not observed. The grave goods indicate cultural contacts of the Kowalewko community with the Elbian circle and the Danish islands.

*(based on the monograph by Tomasz Skorupka: Kowalewko 12. Biritual cemetery of a population of the Wielbark Culture (mid 1st to beginning of 3rd century AD) [Kowalewko 12. Cmentarzysko birytualne ludnosci kutury wielbarskiej (od połowy I w. n.e. do poczatku III w. n.e.)], published in: Marek Chlodnicki [ed.], Archaeological rescue investigations along the gas transit pipeline [Archeologiczne badania wzdluz trasy gazociagu tranzytowego], vol. II - Wielkopolska, part 3, Poznan 2001).*

**Wielbark Culture**

Archaeological culture, deriving its name from an eponymic cemetery discovered at Malbork – Wielbark site, known from today’s Poland (from Pomorze Zachodnie – Western Pomerania through northern Wielkopolska – Greater Poland, Mazowsze – Masovia, Podlasie, up to Lublin region), western Belarus and western Ukraine. Chronology spans almost all the Roman Iron Age, since ca. 20 AD to ca. 450 AD. The Wielbark culture is associated with the Goths and Gepids, who migrated from Scandinavia towards the Black Sea, and their successors, who, after several centuries, returned to the lands formerly occupied by their ancestors. Typical features of the culture include inhumation graves rich in goods of numerous ornaments frequently of noble metals, while no implements and weapons have been observed and iron objects very rarely. Less frequent cremations. Barrows recorded within cemeteries reflect emergence of elites. The Wielbark communities built stone constructions, including pebbled floors and circles. This culture is mainly known from cemeteries, as settlements, not fortified, are less recognized.

**Sample preparation**

Skeletons are the part of the Anthropological Collection at the Institute of Anthropology, Adam Mickiewicz University in Poznan, Poland. 60 well-preserved individuals were selected for the studies (supplementary table S1). From each specimen at least one intact tooth was collected, keeping all the precautions to avoid modern human DNA contaminations.

All pre-PCR procedures were performed in the sterile laboratory dedicated strictly to the studies of aDNA. The laboratory is located at the Faculty of Biology of Adam Mickiewicz University in Poznań, separated from modern DNA laboratories, equipped with automatic UVC lamps, positive air pressure with HEPA filters and divided into rooms dedicated for cleaning and drilling of the samples, and separately for ancient DNA (aDNA) extraction and genomic library preparation.

**Comparative data for population genetic analyses**

For comparative analyses of newly reported individuals living between Oder and Vistula rivers in Iron Age (Kow-OVIA), we used prehistoric and present-day mitochondrial data from published sources.

Prehistoric comparative data

The mtDNA data of Kow-OVIA were compared with published prehistoric data from Europe and Western Eurasia. Those datasets were separated into groups based on cultural, chronological, and geographic features (Supplementary Table S7). Those include: Paleo-Mesolithic Hunter-Gatherers metapopulations: Central/North European Hunter-Gatherers (HGCN), Southwestern European Hunter-Gatherers (HGSW), East European Hunter-Gatherers (HGE). Early European Farmer groups (EEF) and the Neolithic cultures composed of the Starčevo Culture population (STA), Linearbandkeramik in Transdanubia (LBKT), Linearbandkeramik population from Central Europe (LBK), the Neolithic cultures representing temporal succession in Central European region: Rössen Culture (RSC), Schöningen Group (SCG), Baalberge Culture (BAC), Salzmünde Culture (SMC) and Bernburg Culture (BEC); Late Neolithic and Early Bronze Age cultures: Corded Ware Culture (CWC), Bell Beaker Culture (BBC) and Unetice Culture (UC); prehistoric populations from Southern Scandinavia: a Neolithic Hunter-Gatherer population from the Pitted Ware culture (PWC), contemporaneous with it individuals from the Funnel Beaker culture (TRB); individuals from Jutland Iron Age period (JIA); populations of Southwestern European origin: Cardial/Epicardial culture of the Iberian Penisula (CAR), Portuguese Neolithic population (NPO), Neolithic population from Basque Country and Navarre (NBQ), Iberian Chalcolithic El Mirador Cave individuals (MIR), individuals from Iberian Iron Age period (IIA). Additionally, we included population of the Treilles culture from Southeast France (TRE), Early/Middle Neolithic Paris Basin individuals from Gurgy 'Les Noisats' group (RRBP), Bronze Age Kurgan samples from South Siberia (BAS), Bronze Age population from Kazakhstan (BAK), sample set representing steppe herder population of Yamnaya culture (YAM), Iron Age Scythian samples (SCY), individuals from the Scytho-Siberian Pazyryk Culture (SSP). For references, see Supplementary Table S7.

Present-day comparative data

To unravel affinities of Kow-OVIA maternal gene pool to present-day populations, we compared our prehistoric data set to data from extant populations of Europe, Near East, Asia and Africa. The present-day data were pooled into 2 following comparative datasets, that were used as an input for different statistical methods:

1. Central European metapopulation (CEM, n=500), which represents the mtDNA variation in this region. Samples were randomly drawn from a pool of HVS-I sequences from Austria, the Czech Republic, Germany and Poland.
2. Dataset of 73 populations from Europe, Near East, Asia and Africa, used for haplogroup frequency Principal Component Analysis (PCA).

**Analysis of genetic distances - substitution model and gamma value selection**

Depending on the sample composition within each statistical test applied, we evaluated the most suitable substitution model and an associated gamma value separately for each analysis with the Akaike and Bayesian information criterion (AIC and BIC) in jModelTest 2.0 v.0.1.1. Genetic distances between CEPT populations were computed with Tamura & Nei substitution model and with an adjusted gamma-value of 0.312. Fst values between EPT populations were computed with Tamura & Nei substitution model and with an adjusted gamma-value of 0.3420.

AMOVA analyses were performed in Arlequin 3.5.1 using Tamura & Nei substitution model and a gamma value of 0.2650 (without CEM) or 0.312 (with CEM).


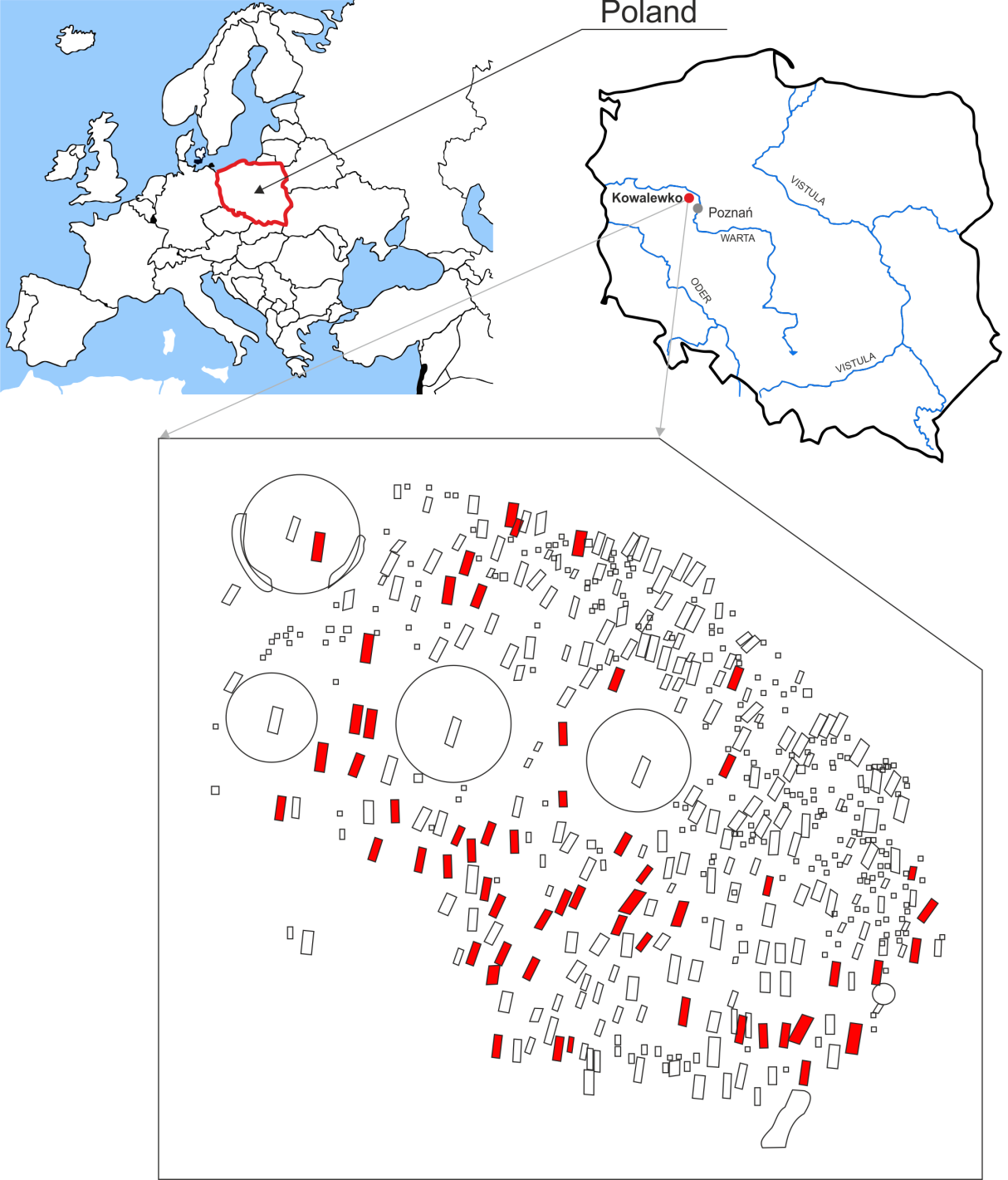


**Supplementary Figure S1:** Location of Kowalewko and a scheme of the Kowalewko cemetery site 12, based on the Fig. 3 from the monograph by Tomasz Skorupka, Kowalewko 12. Biritual cemetery of a population of the Wielbark Culture (mid 1st to beginning of 3rd century AD), published in: Marek Chlodnicki [ed.], Archaeological rescue investigations along the gas transit pipeline, vol. II - Wielkopolska, part 3, Poznan 2001, generated using Corel Draw ver. 12.0, with the author permission. Sampled graves are marked with a red color. Europe and Poland maps were downloaded from Wikimedia Commons (https://commons.wikimedia.org), under the free licence, and modified with Corel Draw ver. 12.0.


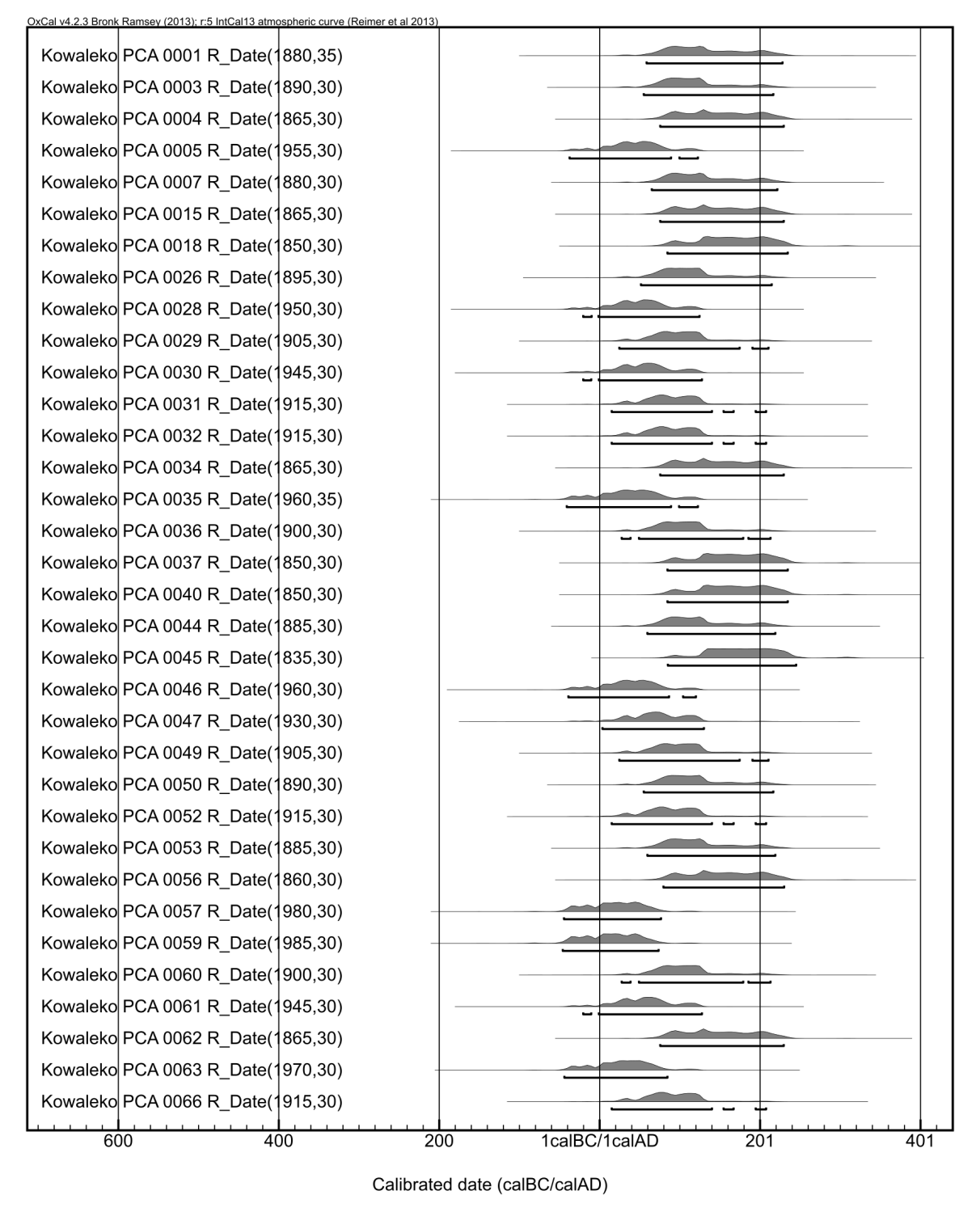


**Supplementary Figure S2:** Results of radiocarbon (^14^C) dating of Kow-OVIA individuals, supplied by the Poznan Radiocarbon Laboratory.


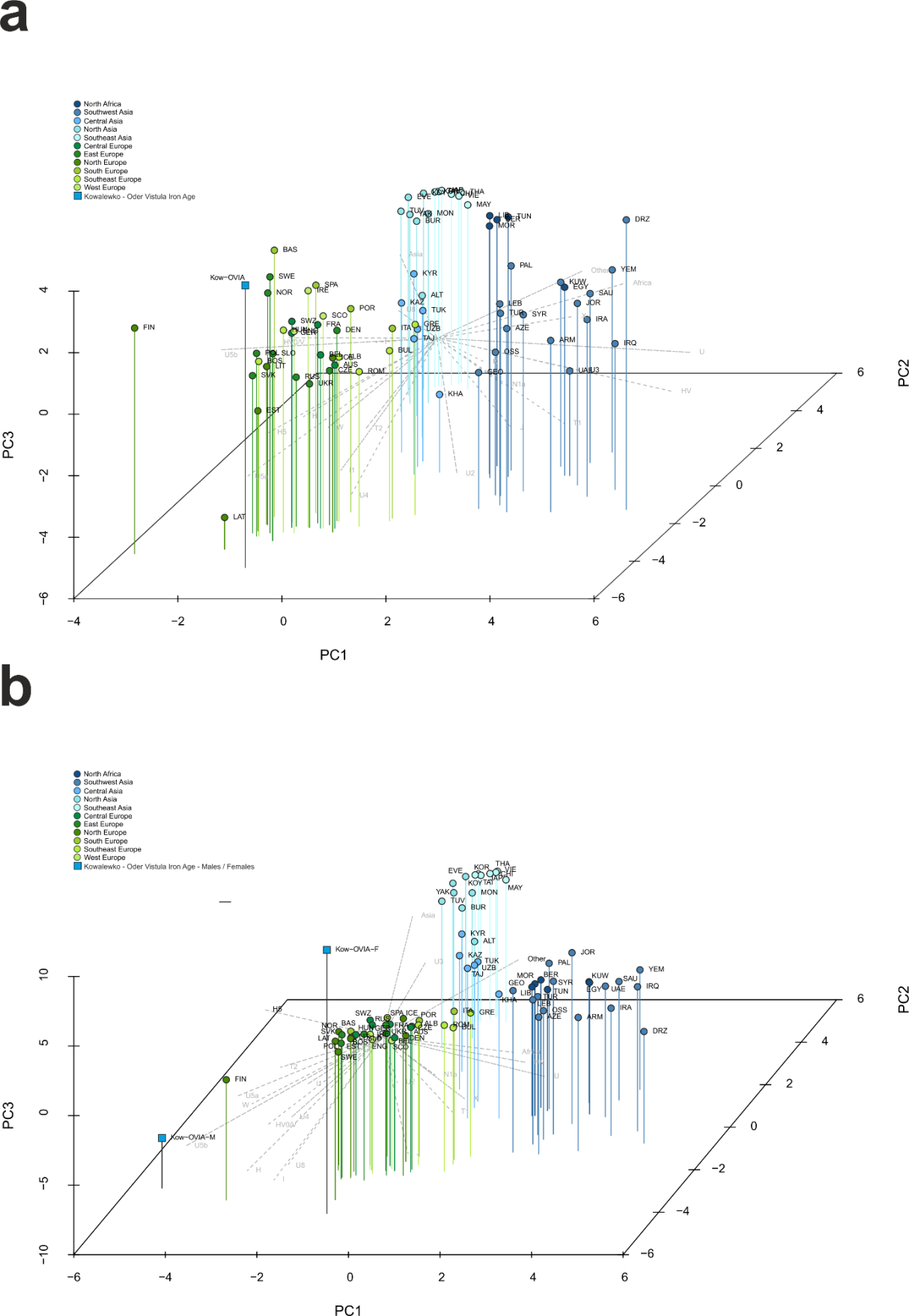


**Supplementary Figure S3:** (a) 3D PCA on haplogroup frequencies of Kow-OVIA and 73 present day populations from Europe and the Near East. (b) 3D PCA on haplogroup frequencies of the same set of populations, with Kow-OVIA divided into female (Kow-OVIA-F) and male (Kow-OVIA-M) subgroups.

**
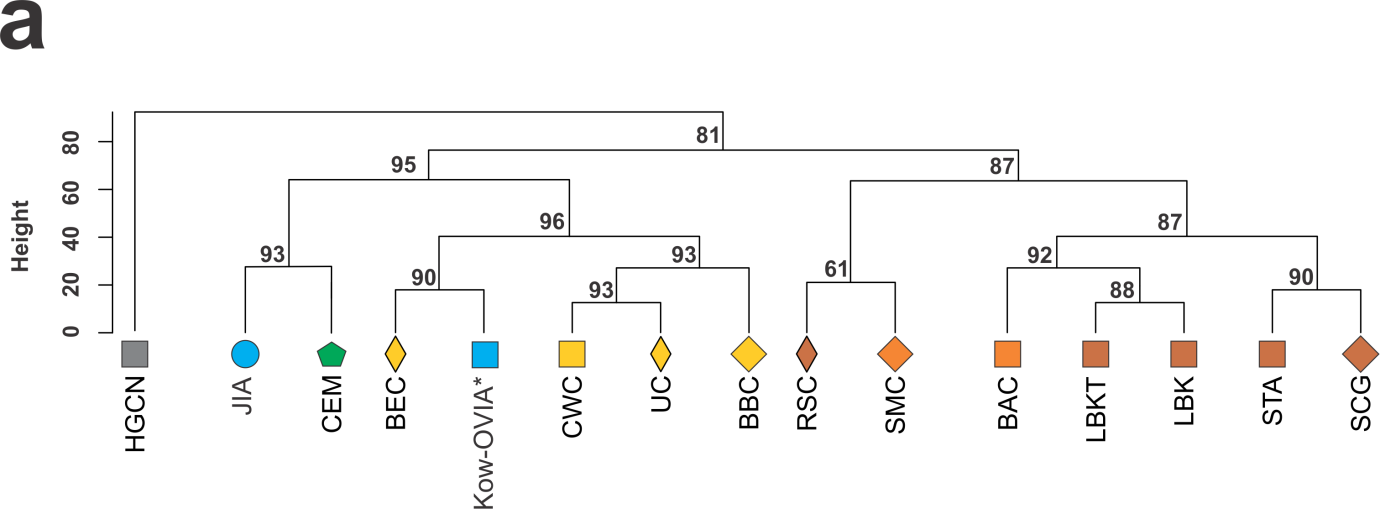
**

**Supplementary Figure S4:** Unsupervised hierarchical clustering with the Ward method and Euclidean distance on haplogroup frequencies for the CEPT populations, where Kow-OVIA* contains potentially maternally related individuals. P-values of the clusters are given as the percent of reproduced clusters based on 10,000 bootstrap replicates.

**
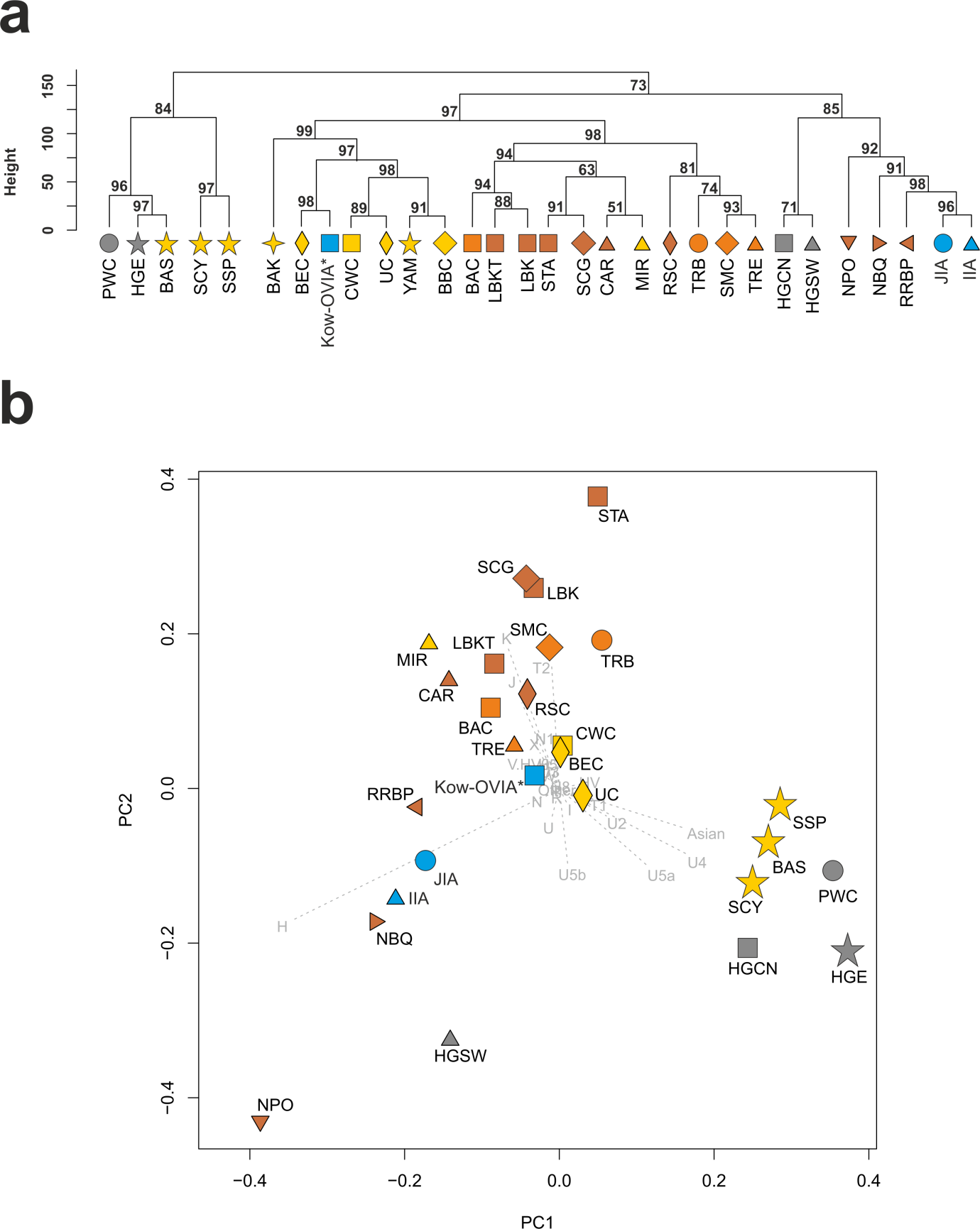
**

**Supplementary Figure S5:** (a) Unsupervised hierarchical clustering with the Ward method and Euclidean distance on haplogroup frequencies for the EPT populations. P-values of the clusters are given as the percent of reproduced clusters based on 10,000 bootstrap replicates. (b) PCA on the haplogroup frequencies of EPT populations. Kow-OVIA* contains potentially maternally related individuals.

**
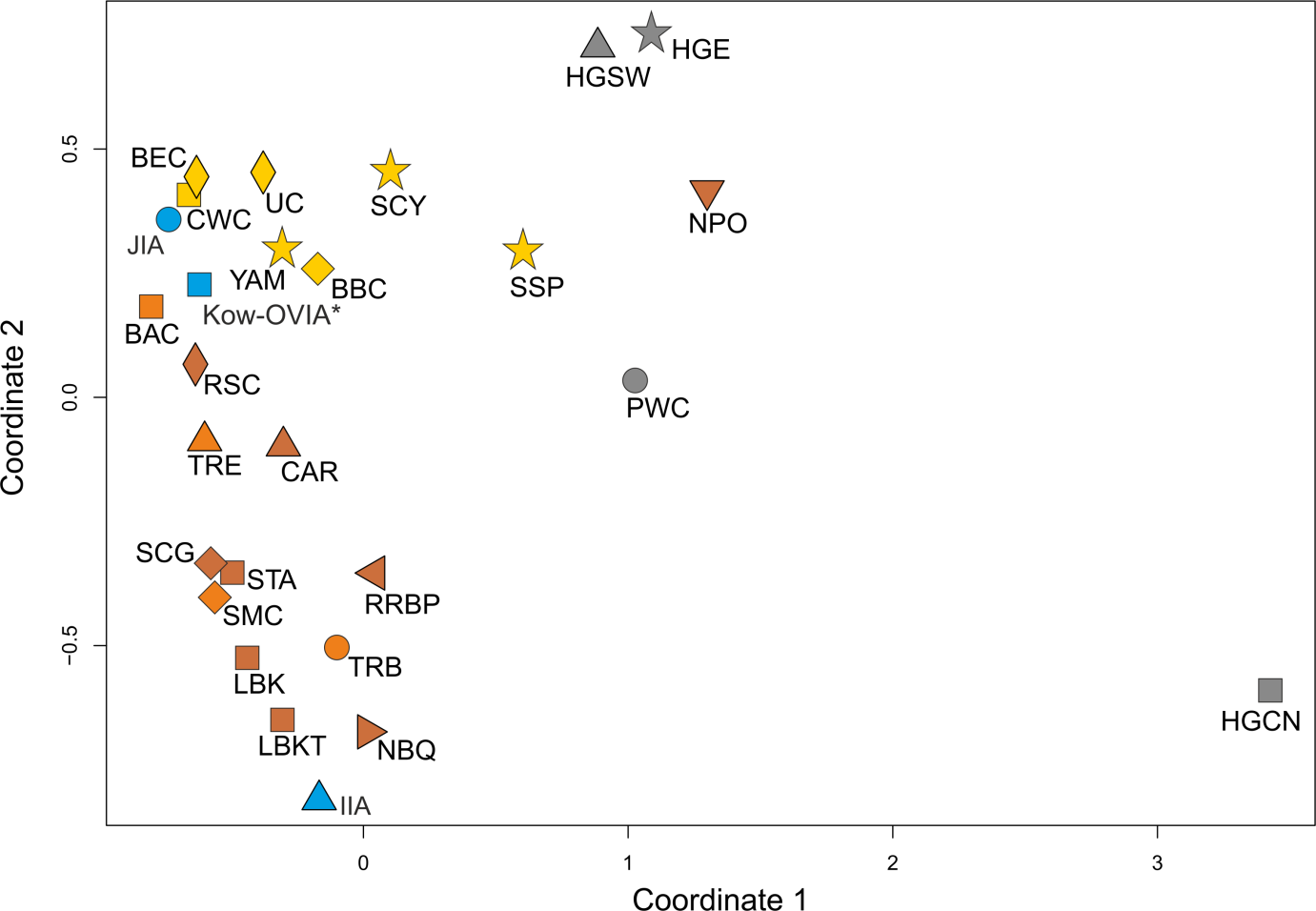
**

**Supplementary Figure S6:** MDS plot of Slatkin’s Fst values for EPT populations. Fst values were obtained for mtDNA HVS-I region (16064-16400 np). Symbols and color shading as in Fig. 2.

**
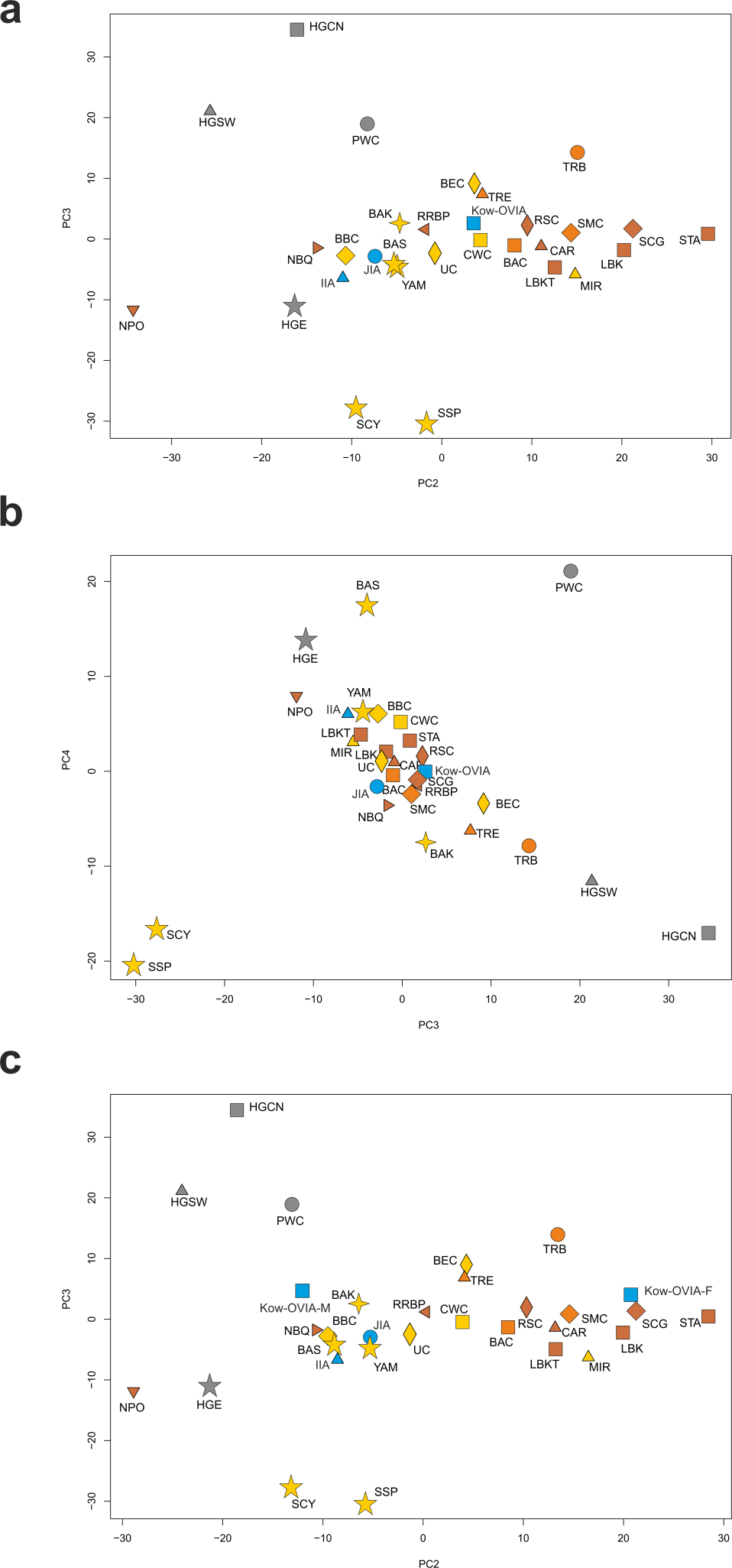
**

**Supplementary Figure S7:** PCAs on the haplogroup frequencies of EPT populations with plotted (a) PC2 and PC3, (b) PC3 and PC4.
